# SIRT1 is a positive regulator of the master osteoblast transcription factor, RUNX2

**DOI:** 10.1371/journal.pone.0178520

**Published:** 2017-05-25

**Authors:** Kayvan Zainabadi, Cassie J. Liu, Leonard Guarente

**Affiliations:** Glenn Center for the Science of Aging, Department of Biology, Koch Institute, MIT, Cambridge, Massachusetts, United States of America; Kyungpook National University School of Medicine, REPUBLIC OF KOREA

## Abstract

Activation of SIRT1 has previously been shown to protect mice against osteoporosis through yet ill-defined mechanisms. In this study, we outline a role for SIRT1 as a positive regulator of the master osteoblast transcription factor, RUNX2. We find that *ex vivo* deletion of *sirt1* leads to decreased expression of *runx2* downstream targets, but not *runx2* itself, along with reduced osteoblast differentiation. Reciprocally, treatment with a SIRT1 agonist promotes osteoblast differentiation, as well as the expression of *runx2* downstream targets, in a SIRT1-dependent manner. Biochemical and luciferase reporter assays demonstrate that SIRT1 interacts with and promotes the transactivation potential of RUNX2. Intriguingly, mice treated with the SIRT1 agonist, resveratrol, show similar increases in the expression of RUNX2 targets in their calvaria (bone tissue), validating SIRT1 as a physiologically relevant regulator of RUNX2.

## Introduction

The mammalian genome contains seven yeast SIR2 homologues which have been named the Sirtuins. SIRT1 is the mammalian orthologue of SIR2 and is an NAD+ dependent deacetylase that plays a key role in regulating pathways ranging from metabolism to aging [1–2]. Cementing its important role in mammalian longevity, mice genetically engineered to overexpress *sirt1*, or wildtype mice treated with SIRT1 agonists, show increased healthspan [3–11] and lifespan [12–14]. These increases are associated with a delay in the onset of many aging-related diseases, including osteoporosis [5–6, 14]. How SIRT1 helps preserve bone mass during aging is not clearly understood.

Osteoporosis is a classic aging disease associated with low bone mass that arises when bone remodeling (the coupled process of bone formation by osteoblasts and resorption by osteoclasts) becomes uncoupled [15]. Type 2 osteoporosis (also known as aging-related osteoporosis) occurs in both sexes and is associated with decreased bone formation by osteoblasts. In contrast, Type 1 osteoporosis (or post-menopausal osteoporosis) results primarily from increased osteoclast activity due to waning circulating estrogen levels (an osteoclast inhibitor) following menopause.

Osteoblasts are derived from a pluripotent mesenchymal stem cell (MSC) population in the bone marrow that also gives rise to adipocytes, myocytes, and chondrocytes [16]. Differentiation of MSCs down any lineage involves at least two steps: first, commitment to a particular fate *(ie* from MSC to an osteoblast progenitor); and second, differentiation of the progenitor cell to a terminal cell type *(ie* from pre-osteoblast to osteoblast). How a pluripotent stem cell commits and then differentiates into a mature osteoblast involves a complex circuitry of external and internal cues consisting of both pro/anti-stimulatory signals [17].

One of the key pro-osteoblast factors is the RUNX2 transcription factor. RUNX2 has been shown to be essential for osteoblast differentiation and bone formation–*runx2* knockout mice lack a mineralized skeleton, and overexpression of *runx2* is sufficient to activate the osteoblast transcriptional program [18–20]. The early induction of RUNX2 expression and activity occurs in part by the homeodomain transcriptional regulators: MSX2, DLX3 and DLX5 [17, 21–24]. Further, the adipocyte master transcription factor, PPARγ—which SIRT1 is a known repressor [25]—has been reported to repress RUNX2 activity and thereby direct the MSC precursor cell away from the osteoblast and towards the adipocyte lineage [26–27]. Additionally, RUNX2 has also been reported to be regulated at a post-translational level via phosphorylation, ubiquitination, and acetylation in response to different stimuli [28–31].

Once the RUNX2 transcriptional program is established, RUNX2 mediates its effects by binding to osteoblast specific cis-acting elements (OSE2) in the promoter of nearly all of the major osteoblast genes [18, 32–33]. Many of these genes are extracellular matrix protein necessary for mineralization and bone formation, including *osteocalcin*, *osteopontin*, and *bone sialoprotein* (BSP). Another target of RUNX2, *osterix*, is a second transcription factor that is essential for the establishment of the late osteoblast program, including mineralization [34–35]. Highlighting its importance, *osterix* knockout mice also fail to develop a mineralized skeleton [35].

Here we present evidence that SIRT1 interacts with and regulates the transcriptional activity of RUNX2. This regulation has important consequences: osteoblast cells lacking SIRT1 show decreased differentiation whereas cells treated with SIRT1 agonists show enhanced differentiation. Interestingly, mice fed resveratrol, another SIRT1 agonist, also show evidence of increased RUNX2 activity in their calvaria (bone tissue), indicating that this regulation is physiologically relevant.

## Materials and methods

### Animal experimentation

All mice were housed under controlled temperature (25 ± 1°C) and lighting conditions and fed standard chow unless otherwise indicated. Sirt1^flox^*/*^flox^ mice were obtained from Jackson Laboratory. All mice were cared for in accordance with the MIT Committee on Animal Care (MITCAC) which approved this study.

For resveratrol feeding experiments, 12 month old male C57BL/6J mice were fed 400mg/kg/day resveratrol or vehicle control in standard chow. After four months, mice were euthanized via carbon dioxide asphyxiation as approved by MITCAC and the calvaria (top of the skull) isolated, washed extensively to remove non-osseous tissue and flash-frozen in liquid nitrogen. For RNA isolation, calvaria was minced in Trizol (Thermo Fisher), and thoroughly homogenized using a Tissue Tearor homogenizer (VWR). Lysates were then spun down at 15,000g for 10 minutes, with the resulting supernatant used for RNA isolation using the RNeasy MinElute Cleanup kit (Qiagen).

### Isolation and differentiation of primary osteoblasts

Primary osteoblast precursors were isolated from 1–3 day old pups as previously described [36]. Calvaria were removed, cleaned and placed in 5mL of no-serum α-MEM media (Sigma Aldrich) containing of 0.1% Collagenase type 2 (Sigma Aldrich) and 0.1% Trypsin/EDTA (Sigma Aldrich). Eight serial incubations were performed at 37°C for 15 minutes in an orbital shaker, with the solutions from the first two incubations discarded, and the remaining solutions combined for plating in α-MEM with 10% FBS (3 pups/10cm plate). Cells were expanded for a maximum of three passages, then trypsinized, filtered through a 70μm nylon filter (BD Falcon), and plated in 6 or 12-well plates for experiments.

Cells were allowed to reach confluency, and two days thereafter infected with either CRE-GFP or empty vector adenovirus (Viral Vector Core Facility, University of Iowa) at 50 multiplicity of infection (MOI) for 24 hours in α-MEM containing 10% FBS. Cells were then allowed to recover for 24 hours before being differentiated with 50μg/mL ascorbic acid and 10mM β-glycerophosphate (Sigma Aldrich). Cells were stained for alkaline phosphatase (3–6 days post-differentiation) using the Alkaline Phosphatase Blue Membrane Substrate Kit (Sigma Aldrich) and mineralization (6–12 days post-differentiation) using 1% Alizarin Red (VWR). Alkaline phosphatase activity was ascertained using p-Nitrophenyl Phosphatase Liquid Substrate System (Sigma Aldrich). In experiments with SRT1720 and SRT2183 (SirTris), drugs were added at a final concentration of 1μM (unless otherwise stated) at Day 0 and DMSO was used as empty vehicle control.

### Quantitative reverse-transcription polymerase chain reaction (qRT-PCR)

Total RNA was extracted from cells (3–6 days post-differentiation) or tissues using Trizol (Thermo Fisher) and the RNeasy MinElute Cleanup kit (Qiagen). 1μg of RNA was used for cDNA synthesis using the SuperScript III reverse transcriptase kit (Thermo Fisher). cDNA was then subjected to qRT-PCR analysis with gene-specific primers in the presence of iQ-SYBR green (Bio-Rad) (Table 1). At least three biological replicates and three technical replicates were used for quantitation of relative mRNA abundance after normalization to ribosomal *rpl19* levels.

**Table 1:** Primer sequences used in this study.

Gene	Sequence
Runx2 type II	*forward*: TGA GAT TTG TGG GCC GGA
	*reverse*: TCT GTG CCT TCT TGG TTC CC
Runx2 type I	*forward*: ATG CGT ATT CCT GTA GAT CCG AGC
	*reverse*: GGT GGT CCG CGA TGA TCT
Osteocalcin	*forward*: AAG CAG GAG GGC AAT AAG GT
	*reverse*: TTT GTA GGC GGT CTT CAA GC
Osterix	*forward*: GCA AGG CTT CGC ATC TGA AA
	*reverse*: AAC TTC TTC TCC CGG GTG TGA
Bone Sialoprotein	*forward*: CAG GGA GGC AGT GAC TCT TC
	*reverse*: AGT GTG GAA AGT GTG GCG TT
Osteopontin	*forward*: AGC AAG AAA CTC TTC CAA GCA A
	*reverse*: GTG AGA TTC GTC AGA TTC ATC CG
Msx2	*forward*: GGG TCT AAA GCG GAA GTC ACT
	*reverse*: GAT GGC GAC CAC TTT CTT GTT
Dlx5	*forward*: TCT CTA GGA CTG ACG CAA ACA
	*reverse*: GTT ACA CGC CAT AGG GTC GC
Dlx3	*forward*: CAC TGA CCT GGG CTA TTA CAG C
	*reverse*: GAG ATT GAA CTG GTG GTG GTA G
AP2	*forward*: GGG GCC AGG CTT CTA TTC C
	*reverse*: GGA GCT GGG TTA GGT ATG GG
PPARγ	*forward*: TCG CTG ATG CAC TGC CTA TG
	*reverse*: GAG AGG TCC ACA GAG CTG ATT
Lipoprotein Lipase	*forward*: GGG AGT TTG GCT CCA GAG TTT
	*reverse*: TGT GTC TTC AGG GGT CCT TAG
Rpl19	*forward*: AAG CCT GTG ACT GTC CAT TC
	*reverse*: CTT CTT GGA TTC CCG GTA TC

### Luciferase assays

For RUNX2 luciferase assays, the U2OS osteosarcoma cell line (ATCC) was used owing to its high transfection efficiency (primary osteoblasts proved difficult to transfect) and its previous use in RUNX2 luciferase studies [37–39]. U2OS cells were grown to 50% confluency and then transfected (Fugene HD, Roche) with the RUNX2 luciferase reporter construct (p6OSE2) with Renilla as an internal control [32–33]. To measure the effect of SIRT1, cells were transfected with either SIRT1 overexpression constructs: pBABE-Vector or pBABE-SIRT1; or SIRT1 RNAi constructs: pSUPERretro-Vector or pSUPERretro-SIRT1 [25]. SIRT1 agonists, SRT1720 and SRT2183 (SirTris) [40], were added at a final concentration of 1μM and DMSO was used as empty vehicle control. Luciferase activity was measured 24 hours after transfection (or 4–6 hours after addition of drugs) from four biological replicates according to manufacturer instructions using a dual luciferase reporter kit from Promega.

### Western blot and immunoprecipitation

Antibodies for western blotting and immunoprecipitation (IP) were obtained from the following sources: SIRT1 (Upstate), RUNX2 (Sigma and Abcam), HSP90 (Abcam), SIRT6 (Cell Signaling), FLAG (Sigma Aldrich), HA (Santa Cruz Biotechnology). IPs were carried out in the following manner: 293T cells were transfected with FLAG-SIRT1, HA-RUNX2 or both, with empty plasmid backbones serving as controls.

For endogenous IPs, U2OS osteosarcoma cells (ATCC) were used due to their fast replicative cycle, which provided ample starting material for performing and optimizing IP conditions. 15cm plates were washed twice with phosphate buffered saline (PBS), removed in the presence of PBS + 0.5% Triton X100 (Sigma Aldrich) and complete protease inhibitors (Roche), and homogenized by passing (5 times each) through a 21G, 23G, and finally a 26G needle. Cells were then allowed to lyse on an orbital shaker for 30 minutes (4°C) and then centrifuged at 15,000g for 10 minutes (4°C). The resulting supernatants were used for IP experiments. IPs were generally performed using antibodies at a final concentration of 0.5ug-1μg/ml with incubation performed overnight at 4°C. Protein G agarose (Santa Cruz Biotechnology) was then added for 1–2 hours, after which the agarose was washed at least 3 times with PBS + 0.1% Triton X100, and then boiled for 3 minutes in SDS sample buffer. The IP eluates were run on 4–15% gels (Bio-Rad), transferred to nitrocellulose membrane, and probed by Western blot.

### Statistical analysis

Statistical analysis was performed using an unpaired Student’s t test, and significant differences are indicated by single asterisk (*) when p < 0.05, double asterisk (**) when p < 0.01, and tripe asterisk (***) when p<.005. All data is presented ± standard error of the mean (SEM). Experiments were performed at least two independent times (with the exception of the *in vivo* resveratrol feeding experiments) to ensure reproducibility.

## Results

### Deletion of SIRT1 inhibits osteoblast differentiation

**Fig 1:**
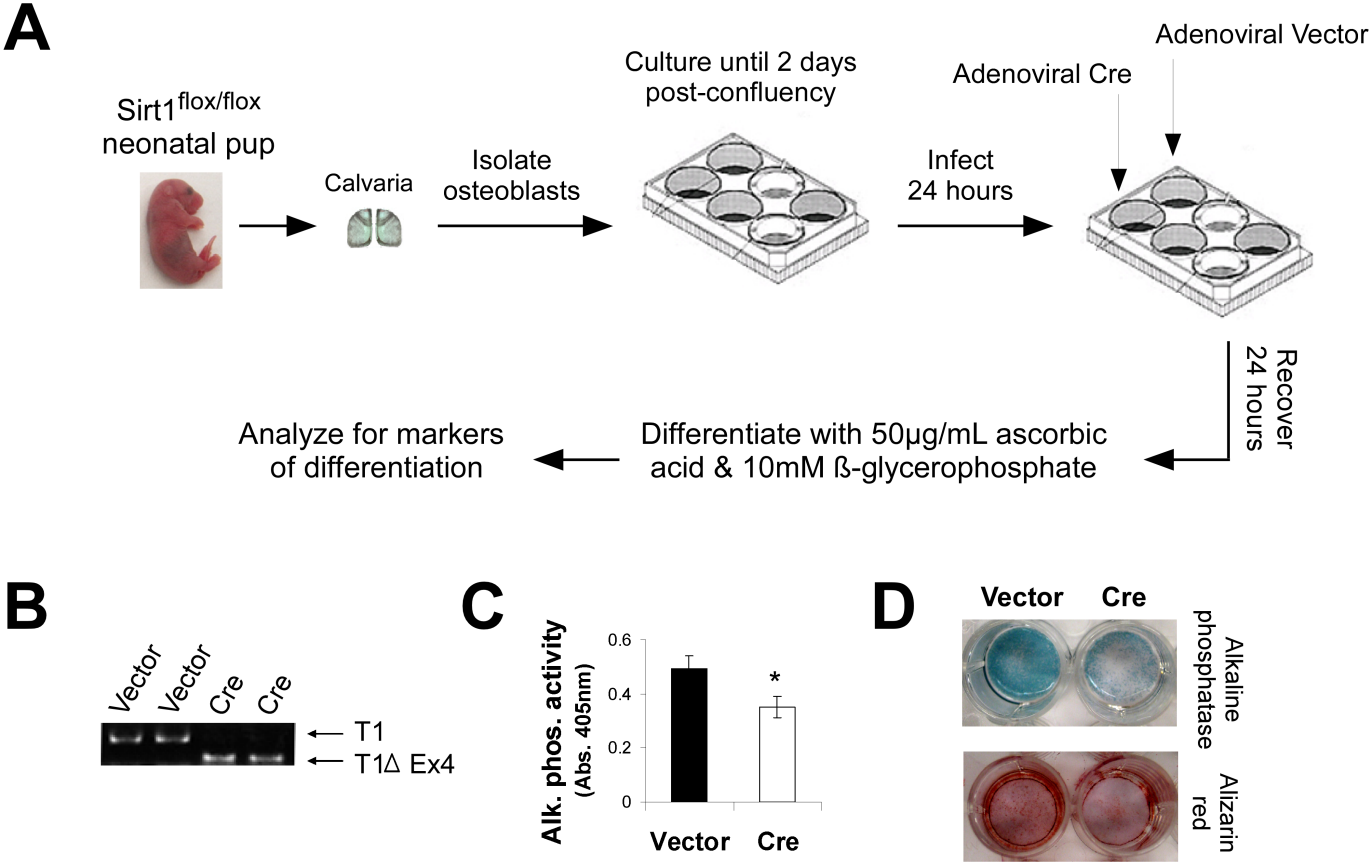
*Ex vivo* deletion of *sirt1* inhibits osteoblast differentiation. **A)** Primary osteoblasts obtained from the calvaria of Sirt1^flox^*/*^flox^ neonates were infected two days post-confluency with adenoviral-CRE to excise *sirt1*. **B)** Cells infected with adenoviral-CRE show excision of SIRT1 catalytic exon 4 (T1Δ4) as indicated by a smaller PCR product obtained with primers flanking exon 4. **C)** CRE-infected cells show reduced alkaline phosphatase enzymatic activity, an early marker of osteoblast differentiation. (n = 3, * p<.05) **D)** CRE-infected cells showed reduced staining for two different markers of osteoblast differentiation, alkaline phosphatase and alizarin red, a marker of mineralization.

To determine how SIRT1 regulates osteoblast differentiation, we isolated primary osteoblasts from Sirt1^flox^*/*^flox^ neonatal mice and excised SIRT1 *ex vivo* with the use of adenoviral CRE (Fig 1A). To minimize any extraneous effects of SIRT1 on cell proliferation and/or terminal cell division (as opposed to differentiation *per se*), cells were infected two days post-confluency. Upon addition of CRE-adenovirus, the *sirt1* catalytic domain (exon 4) is excised with near 100% efficiency, creating in effect an isogenic SIRT1 knockout cell line (Fig 1B). Osteoblasts deleted for SIRT1 displayed marked reductions in both early and late markers of differentiation, including alkaline phosphatase and mineralization, respectively (Fig 1C and 1D). Consistent with previous reports [41–43], these results indicate that SIRT1 is a positive regulator of osteoblast differentiation.

### Deletion of SIRT1 results in decreased expression of RUNX2 downstream targets

**Fig 2:**
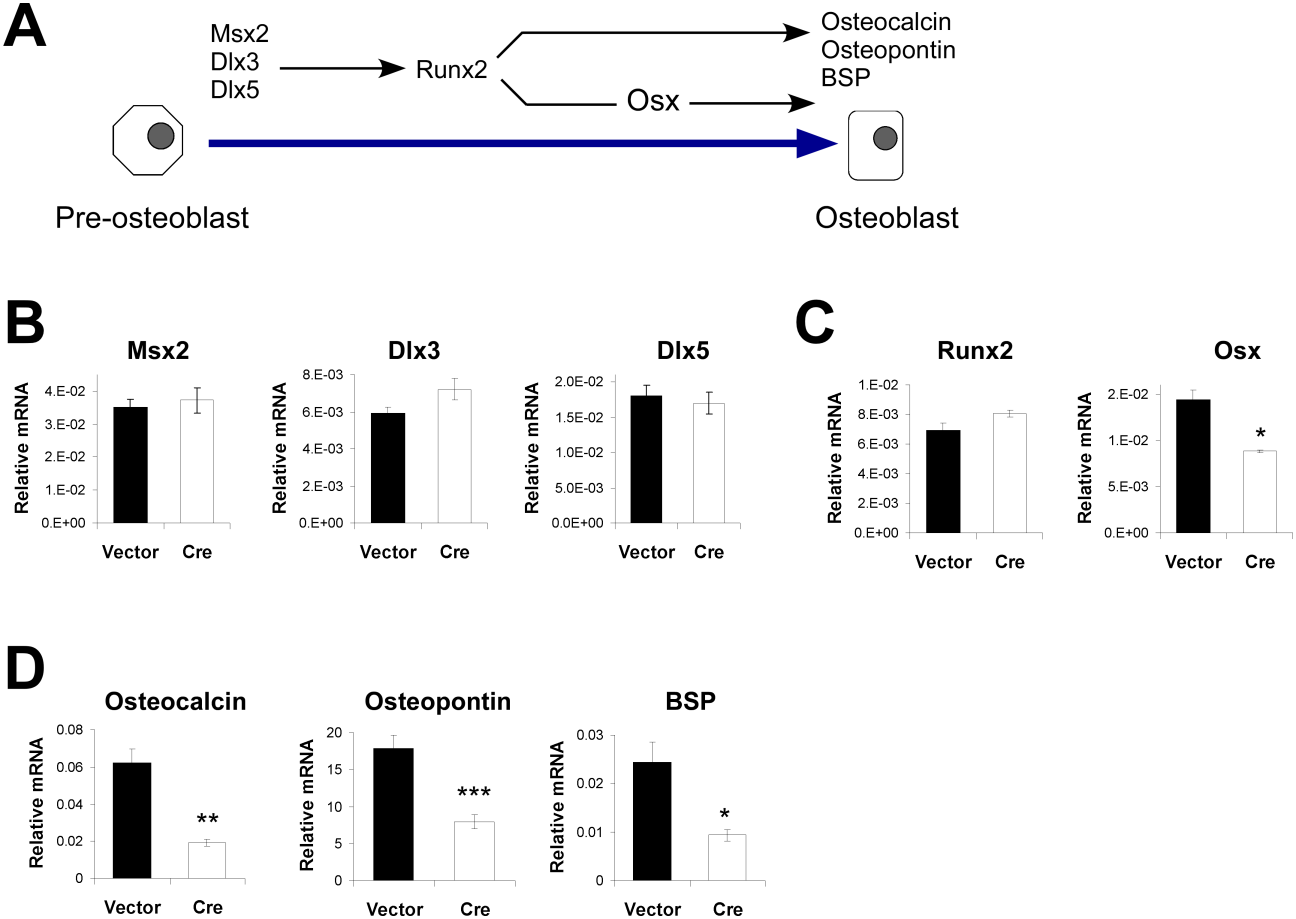
*Ex vivo* deletion of *sirt1* decreases expression of RUNX2 downstream targets. **A)** A schematic representing the osteoblast transcriptional regulators and markers examined in this study. The homeodomain transcriptional regulators, Msx2, Dlx3, and Dlx5, help establish the early osteoblast transcriptional program, including upregulation of Runx2 expression and activity [17, 21–24]. Runx2 then directly binds to and stimulates the transcription of osteoblast specific genes, including Osterix (Osx), an essential osteoblast transcription factor. **B)** There are no differences in the expression of Msx2, Dlx3, and Dlx5 in SIRT1 knockout (Cre-infected) versus wildtype (vector-infected) osteoblasts as ascertained by quantitative reverse-transcription PCR (qRT-PCR). **C)** While SIRT1 knockout osteoblasts (Cre) express comparable amounts of Runx2, they show a near two-fold reduction in the expression of the Runx2 downstream target, Osterix (Osx). **D)** Three other RUNX2 targets, Osteocalcin, Osteopontin, and Bone Sialoprotein (BSP), also show reduced expression in SIRT1 knockout cells (Cre), suggesting decreased transcriptional activity of RUNX2 in the absence of SIRT1. (n≥3, * p<.05; ** p<.01; *** p<.005).

To determine how SIRT1 exerts its effect, we examined the expression of a number of key osteoblast transcription factors (Fig 2A). SIRT1 wildtype and knockout cells show no differences in the expression of the homeobox family of transcriptional regulators, *dlx3*, *dlx5*, and *msx2*, which are important for establishing the early osteoblast transcriptional program [17, 21–24] (Fig 2B). Expression of the master osteoblast transcription factor, *runx2*, is also unchanged (Fig 2C). However, deletion of SIRT1 results in a two-fold reduction of the RUNX2 downstream target, *osterix* (osx), a transcription factor essential for osteoblast differentiation (Fig 2C). Other RUNX2 downstream targets, including *osteocalcin*, *osteopontin*, and *bone sialoprotein* (bsp), also show reduced expression in SIRT1 knockout cells. These results indicate RUNX2 hypoactivity in the absence of SIRT1 (Fig 2D).

SIRT1 is a known repressor of PPARγ, a master adipocyte transcription factor which has previously been shown to repress RUNX2 activity in mesenchymal stem cells [25–26]. We therefore examined the expression of PPARγ and its downstream targets, *lipoprotein lipase* and *adipocyte protein 2* (ap2), as a measure of PPARγ activity. PPARγ and its downstream targets were all undetectable in both wildtype and SIRT1 knockout cells, indicating that PPARγ hyperactivity was unlikely the cause of the observed decreases in RUNX2 transcriptional activity.

### SIRT1 interacts with RUNX2

**Fig 3:**
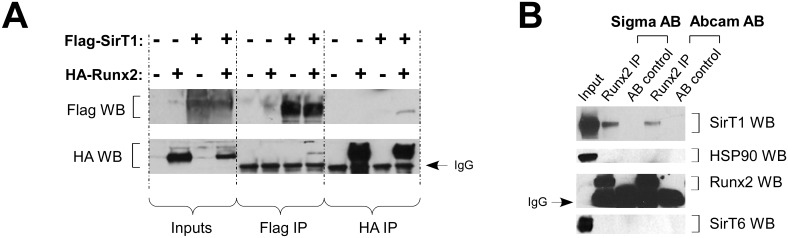
SIRT1 interacts with RUNX2. **A)** Tagged versions of SIRT1 and RUNX2 interact in 293T cells: FLAG-tagged SIRT1 is able to co-immunoprecipitate HA-tagged RUNX2, and vice versa. (WB: western blot; IP: immunoprecipitation) **B)** This interaction also exists at the endogenous level. Two different RUNX2 antibodies (Sigma and Abcam) co-immunoprecipitate SIRT1, but not closely related SIRT6 or abundantly expressed HSP90, in U2OS osteosarcoma cell lysates. The band below the RUNX2 band (in the Runx2 WB panel) represents heavy chain IgG. (AB: antibody).

Since RUNX2 expression itself was not changed, but expression of its downstream targets were, we hypothesized SIRT1 might act in a post-translational manner to increase RUNX2 activity. This would require for SIRT1 and RUNX2 to interact. Co-immunoprecipitation (co-IP) experiments with tagged versions of SIRT1 and RUNX2 show that they do in fact interact: FLAG-SIRT1 was able to co-immunoprecipitate HA-RUNX2, and vice versa (Fig 3A).

To determine whether SIRT1 and RUNX2 interact at endogenous levels in osteoblasts, we first pre-screened a number of commercially available antibodies for their ability to IP and/or detect RUNX2 by Western blot. While none of the antibodies were able to detect endogenous RUNX2 by Western blot, two were able to successfully immunoprecipitate RUNX2 from whole-cell lysates of U2OS osteosarcoma cells (Fig 3B). Importantly, both antibodies also co-immunoprecipitated SIRT1, but not the closely related SIRT6 nor the abundantly expressed HSP90 (Fig 3B). These results indicate that SIRT1 and RUNX2 interact in osteoblasts and that this interaction is specific.

### SIRT1 promotes RUNX2 transcriptional activity

**Fig 4:**
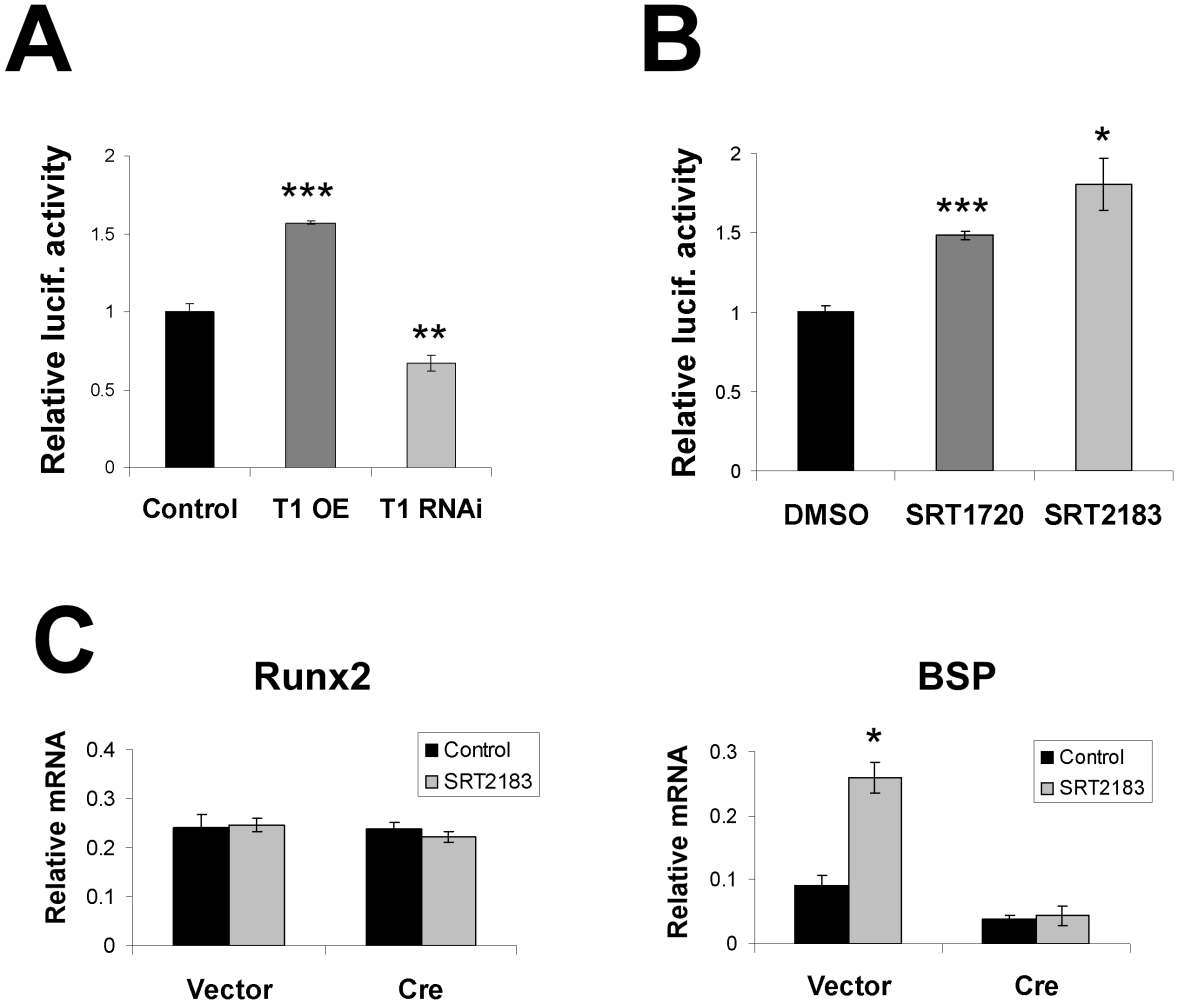
SIRT1 increases the transcriptional activity of RUNX2. **A)** A RUNX2 luciferase reporter assay (p6OSE2) shows that overexpression (OE) of SIRT1 increases luciferase activity, while RNA-interference (RNAi) of SIRT1 decreases it (n = 4). **B)** Two specific SIRT1 activators, SRT1720 and SRT2183, also increase luciferase activity (n = 4). **C)** SIRT1 activator, SRT2183, leads to induction of endogenous RUNX2 target Bone Sialoprotien (BSP) in wildtype (vector) but not SIRT1 excised (Cre) cells, indicating that the stimulatory effects of SRT2183 on RUNX2 is SIRT1-dependent. The expression of Runx2 itself is unchanged. (n≥3, * p<.05; ** p<.01; *** p<.005).

Next, to determine the molecular consequences of this interaction, we used a RUNX2 dual luciferase reporter (p6OSE2) which contains six RUNX2 osteoblast specific cis-acting elements (OSE2) upstream of luciferase [32–33]. As expected, we saw a dose-dependent increase in luciferase activity with increased dosage of the construct, confirming that endogenous RUNX2 in U2OS cells is able to activate the promoter. Notably, overexpression of SIRT1 significantly increased this luciferase activity, while RNA-interference of SIRT1 reduced it (Fig 4A). Consistent with a stimulatory role for SIRT1, cells treated with two different SIRT1 small molecule activators, SRT1720 and SRT2183 [40], showed a similar increase in luciferase activity (Fig 4B).

We next set out to determine how activation of SIRT1 affected the expression of endogenous RUNX2 targets in osteoblasts. In line with our previous findings, primary osteoblasts treated with SRT2183 (the more potent of the two SIRT1 activators) show increased expression of the RUNX2 downstream target *bone sialoprotein* (BSP), but not that of *runx2* itself (Fig 4C). This increase in BSP expression was not observed in osteoblasts in which SIRT1 had been deleted, indicating that the effect of SRT2183 on RUNX2 activity was SIRT1 dependent (Fig 4C).

### Activation of SIRT1 stimulates expression of RUNX2 targets *in vivo*

**Fig 5:**
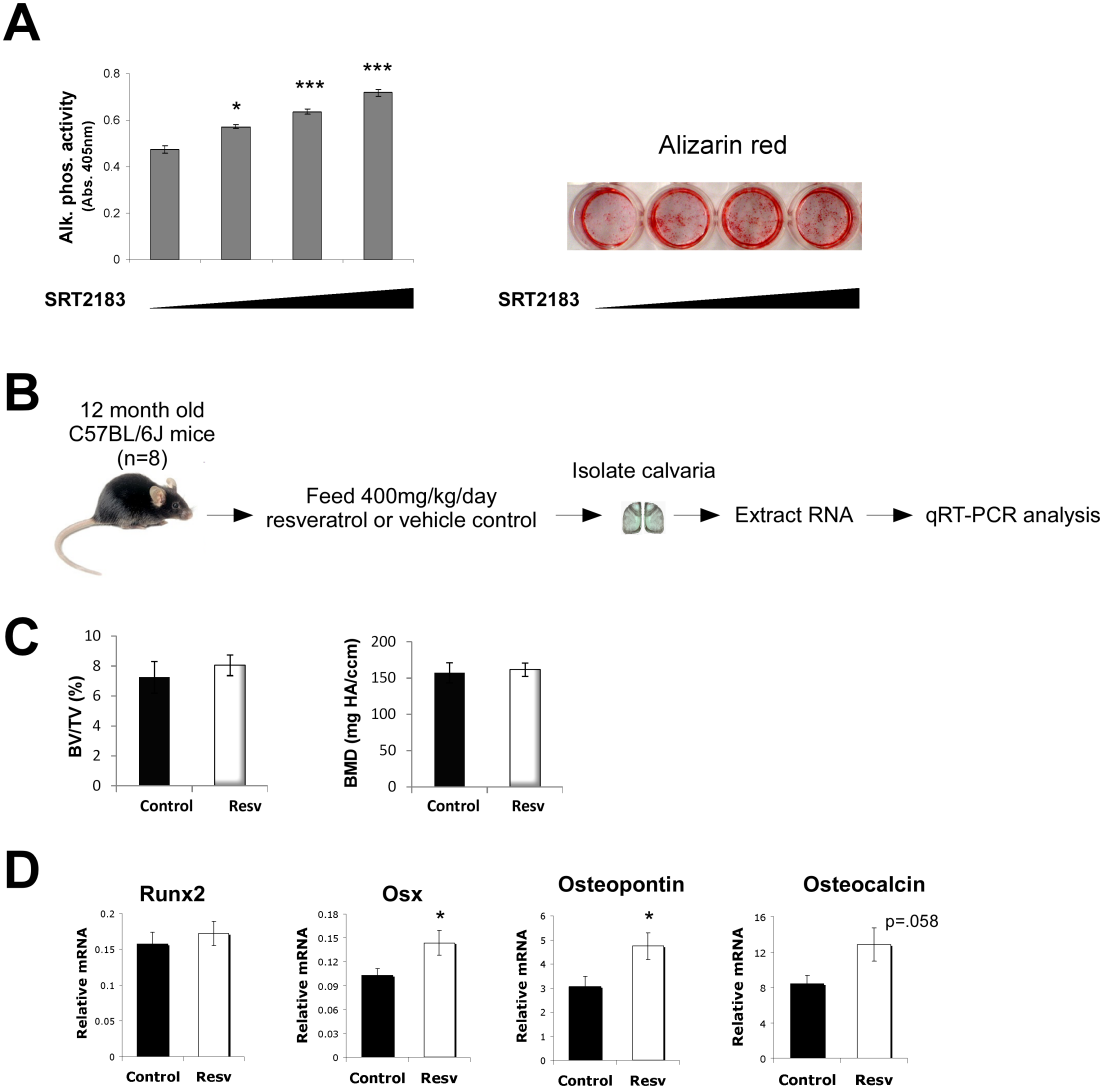
Pharmacological activation of SIRT1 promotes osteoblast differentiation, and expression of RUNX2 targets *in vivo*. **A)** Treatment of primary osteoblasts with SIRT1 activator, SRT2183, increases markers of differentiation (alkaline phosphatase and alizarin red) in a dose-dependent manner. **B**) Mice (n = 8) were fed 400mg/kg/day of resveratrol (another SIRT1 activator) or vehicle control and had the expression of RUNX2 downstream targets examined in their calvaria (skullcap) by qRT-PCR. **C)** Resveratrol (Resv) fed mice show similar bone volume/total volume (BV/TV) and bone mineral density (BMD) as control fed mice (due to the short treatment regimen). **D)** The calvaria of resveratrol fed mice show increased expression of RUNX2 downstream targets, but not RUNX2 itself. (n = 8, * p<.05; ** p<.01; *** p<.005).

We were next interested in determining how pharmacological activation of SIRT1 impacted osteoblast differentiation. Primary osteoblasts treated with SRT2183 showed a dose-dependent increase in markers of differentiation (Fig 5A). These findings are consistent with the observed upregulation of RUNX2 activity and increased expression of OSTERIX.

Intriguingly, another small molecule activator of SIRT1, resveratrol, has been shown to have protective effects against age-related osteoporosis in mice and rats [5, 44]. To determine whether this was in part due to stimulation of RUNX2 activity in bone tissue, we fed mice 400mg/kg/day of resveratrol and then analyzed the expression of RUNX2 targets in their calvaria (skullcap) (Fig 5B). While these mice did not show an increase in bone mass as assessed by microcomputed tomography (due to the short treatment regimen) (Fig 5C), they did intriguingly show similar increases in the expression of RUNX2 downstream targets, but not RUNX2 itself, in their calvaria (Fig 5D). These findings indicate that SIRT1 also acts *in vivo* to stimulate RUNX2 transcriptional activity in bone.

## Discussion

Here, we present evidence that SIRT1 interacts with and positively regulates the transcriptional activity of RUNX2. This regulation has important consequences in osteoblasts: cells lacking SIRT1 show decreased differentiation associated with reduced expression of RUNX2 targets, while cells treated with SIRT1 agonists show enhanced differentiation associated with increased expression of RUNX2 targets (Figs 1 and 2). Importantly, one of these affected downstream targets is OSTERIX (Osx), a second transcription factor essential for osteoblast differentiation [35]. The increase in RUNX2 activity, and resulting increase in OSTERIX expression, is a very plausible explanation for the stimulatory effects of SIRT1 on osteoblast differentiation.

RUNX2 activity has previously been shown to be regulated at a post-translational level, including by class I and class II histone deacetylases. These proteins interact with and repress the activity of RUNX2 [28–30]. SIRT1, a member of the class III histone deacetylases, is therefore unique in that it functions to enhance, and not repress, RUNX2 activity.

The exact mechanism by which SIRT1 regulates RUNX2 activity in osteoblasts is currently unknown, though several lines of evidence point towards its deacetylase activity. First, excision of the catalytic domain of SIRT1 in primary osteoblasts is sufficient to reduce the expression of RUNX2 downstream targets. Conversely, agonists which increase SIRT1 deacetylase activity produce an opposite effect, both in osteoblasts (Fig 4C) and bone tissue in mice (Fig 5D). Whether SIRT1 acts directly on RUNX2 or the histones of RUNX2 targets is yet unknown, though given that histone deacetylation is generally repressive the latter mechanism is unlikely. We thus speculate that it is more likely that SIRT1 deacetylates RUNX2 directly, though we were unable to demonstrate this in the present study. However, a recent report found that treatment with nicotinamide (a pan-Sirtuin inhibitor) or SIRT1 knockdown resulted in increased RUNX2 acetylation in mesenchymal stem cells [45]. It will be important to determine whether a similar mechanism occurs in osteoblasts, and how deacetylation of specific lysine residue(s) affects RUNX2 activity.

Special care was taken to minimize any indirect effects that SIRT1 may have on osteoblast differentiation. This was performed in two ways: 1) First we used primary osteoblasts derived from the calvaria of Sirt1^flox^*/*^flox^ neonates which consist of a relatively pure population of cells already committed to the osteoblast lineage [36]; 2) We excised *sirt1* in these cells two days after they had reached confluency and thus had exited the cell cycle (Fig 1A). An added benefit of such a strategy is that since both wildtype and knockout cells come from the same source (*ie* Sirt1^flox^*/*^flox^ mice) they are essentially isogenic, with the exception of the SIRT1 deletion. These precautions should help reduce any effects SIRT1 might have on cell proliferation/survival, terminal cell division or osteoblast commitment *per se*. This latter point (*ie* commitment versus differentiation) is particularly cogent, as a previous report found an increase in RUNX2 expression (albeit in mesenchymal stem cells, MSCs) upon stimulation of SIRT1 [46]. We did not observe any changes in RUNX2 expression associated with SIRT1 activity, similar to a previous study in osteoblasts [43], which is likely attributable to the fact we used osteoblasts already committed to the osteoblast lineage as opposed to pluripotent MSCs. Consistent with this, PPARγ and its downstream targets were undetectable in our cells but were readily detectable in the MSCs used in the aforementioned study [46].

In contrast, we did find that SIRT1 increased the activity of RUNX2, and the expression of OSTERIX, two pro-osteoblast transcription factors which would be expected to promote differentiation; which was experimentally verified (Fig 5A). Given this stimulatory role in osteoblasts, SIRT1 provides a unique pharmacological target for the treatment of age-related osteoporosis, a disease associated with reduced osteoblast activity (and for which few effective treatments currently exist). Our analysis reveals that pharmacological activation of SIRT1 *in vivo* is associated with upregulation of RUNX2 transcriptional activity in bone tissue (Fig 5D), suggesting this may in part explain the previously described salutary effects of SIRT1 on bone during aging [5, 44]. Intriguingly, a recent randomized placebo-controlled trial in humans showed that resveratrol treatment also led to increased bone mineral density and bone alkaline phosphatase activity in obese men [47].

How this stimulation affects *in vivo* osteoblast differentiation, function and bone formation, and whether RUNX2 or SIRT1 activity in bone change with age are all outstanding questions. Additionally, how *in vivo* activation of SIRT1 affects the other principal cell types of bone, namely osteoclasts and mesenchymal stem cells, is an area of warranted study. Preliminary evidence gives reason to be optimistic: SIRT1 appears to promote commitment of MSCs towards the osteoblast lineage [45–46, 48–49] and repress differentiation of osteoclasts [43, 50–51]. Indeed, recent studies with second and third generation SIRT1 agonists have shown even greater promise in preserving bone mass in mice [14, 52], raising hopes for a possible novel therapeutic for osteoporosis in the not so distant future.
